# High-Pressure Mechanistic Insight into Bioinorganic NO Chemistry

**DOI:** 10.3390/molecules26164947

**Published:** 2021-08-16

**Authors:** Łukasz Orzeł, Maria Oszajca, Justyna Polaczek, Dominika Porębska, Rudi van Eldik, Grażyna Stochel

**Affiliations:** 1 Faculty of Chemistry, Jagiellonian University, Gronostajowa 2, 30-387 Kraków, Poland; lukasz.orzel@uj.edu.pl (Ł.O.); maria.oszajca@chemia.uj.edu.pl (M.O.); justyna.polaczek@uj.edu.pl (J.P.); dominika.porebska@doctoral.uj.edu.pl (D.P.); 2 Department of Chemistry and Pharmacy, University of Erlangen-Nuremberg, Egerlandstr 1, 91058 Erlangen, Germany

**Keywords:** kinetics, high-pressure techniques, nitric oxide, iron complexes, porphyrins, heme proteins, cobalamins

## Abstract

Pressure is one of the most important parameters controlling the kinetics of chemical reactions. The ability to combine high-pressure techniques with time-resolved spectroscopy has provided a powerful tool in the study of reaction mechanisms. This review is focused on the supporting role of high-pressure kinetic and spectroscopic methods in the exploration of nitric oxide bioinorganic chemistry. Nitric oxide and other reactive nitrogen species (RNS) are important biological mediators involved in both physiological and pathological processes. Understanding molecular mechanisms of their interactions with redox-active metal/non-metal centers in biological targets, such as cofactors, prosthetic groups, and proteins, is crucial for the improved therapy of various diseases. The present review is an attempt to demonstrate how the application of high-pressure kinetic and spectroscopic methods can add additional information, thus enabling the mechanistic interpretation of various NO bioinorganic reactions.

## 1. Introduction

With this review, we would like to bring the reader’s attention to high-pressure molecular sciences and technology in the advancement of which Professor Janusz Jurczak made a significant impact, both within synthetic organic chemistry and the development of instrumentation [1,2,3]. Professor Jurczak has been a real leader in this field, and for that reason, the authors of this review dedicate it to his honor. In this review, we focus on the mechanistic studies concerning the interaction of NO with complexes of Fe^II/III^ and Co^II/III^ (including metalloproteins and vitamin B12) performed with the application of high-pressure kinetic techniques. Since this type of study requires specialized equipment, the number of groups dealing with these studies is limited; therefore, herein, we mainly present the studies performed in our group, as well as those that have resulted from our collaboration with other research groups.

Pressure as an experimental variable can provide unique information about the microscopic properties of the studied materials and processes. It can be used as a research tool for investigating the structure, energetics, dynamics, and kinetics of various processes and reactions at the molecular level, as well as to chemical synthesis and modifications of materials to preserve or improve their quality. The pressure range used for such different purposes can extend from a few hundred MPa (reaction kinetics) up to GPa (chemical synthesis or modification of materials). High-pressure kinetic and thermodynamic studies for the elucidation of reaction mechanisms are usually limited to 200 MPa [4,5,6].

In order to come as close as possible to the “real” mechanism, it is essential to gain as much information as possible about the chemical understanding of the studied system, empirical rate law, activation and reaction parameters, evidence for possible intermediates, and theoretical analysis of the suggested mechanism. Among the usual variables used in experimental kinetic studies (solvent, ionic strength, pH, etc.), the temperature is a very useful parameter. From the temperature dependence of rate constants and application of the transition state theory, the determination of the enthalpy of activation (ΔH^‡^) and the entropy of activation (ΔS^‡^)—parameters required for the reaction free energy profile—is possible. The additional application of high-pressure kinetic techniques can reveal crucial mechanistic information for chemical processes through the construction of volume profiles with experimentally determined volumes of activation (ΔV^‡^). Such profiles not only complement free energy profiles for the studied reactions but enable a chemical picture to be visualized on the basis of partial molar volume changes that occur along with the reaction coordinate with the estimation of the reaction volume (ΔV°) and the difference in partial molar volumes of product and reactant species [7,8,9].

From a historical point of view, volumes of activation were obtained for organic reactions long before high-pressure methods were applied to inorganic reactions, for which impressive progress in high-pressure inorganic solution chemistry has been made during the past forty years [10,11]. Furthermore, it was especially the inorganic chemistry community that required experimental techniques to study fast reactions on the second, millisecond, microsecond, and nanosecond timescale. These techniques included stopped-flow, flash-photolysis, pulse-radiolysis, T-jump, and NMR high-pressure developments in the pressure range up to 200 MPa.

In this review, we have selected the bioinorganic chemistry of nitric oxide as an example to show how high-pressure kinetic and thermodynamic studies can assist the elucidation of inorganic reaction mechanisms on the molecular level and to improve our understanding of the processes significant for catalysis, health, and the environment. 

Small inorganic redox molecules, such as nitric oxide (NO), dioxygen (O_2_), or hydrogen sulfide (H_2_S), due to their involvement in numerous catalytic, environmental, and biological processes, have been of interest to scientists from various fields of molecular sciences. Among others, all three molecules and their reactive derivatives play diverse roles in various aspects of cell biology and physiology, including metabolism, cellular signaling, and host defense. The bioinorganic chemistry of these redox molecules plays a central role in their biological activity [12,13,14].

Nitric oxide (NO) is a short-living free radical, and, in contrast to many signaling agents (e.g., various peptides), which rely on receptors where structural relationships determine their function, the chemistry of NO determines its biological roles. There are two distinct reaction types—direct and indirect—which depend on the NO concentration, reactive species formed and reaction kinetics. The direct effects involve interactions of NO itself with biological targets such as redox metal centers, redox-active amino acids, or other radical species. The indirect activity of nitric oxide is mediated by various reactive nitrogen species (RNS). Recognition of the molecular mechanisms for various reaction types by which NO and other RNS can fulfill their biological function is crucial not only for a better understanding of many natural processes but also for planning innovative strategies in health and environmental protection [15,16,17].

## 2. High-Pressure Kinetic Techniques Applied to Mechanistic Studies in Coordination Chemistry

**Scheme 1:**
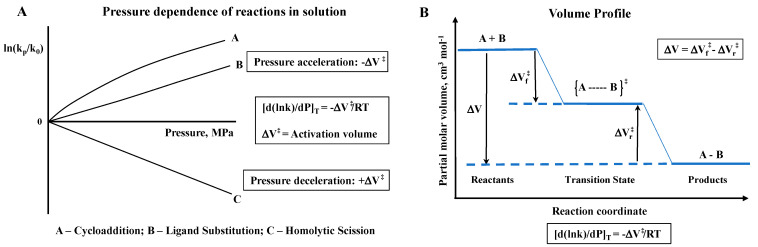
(**A**) Schematic presentation of the dependence of ln(k_p_/k_0_) vs. pressure. (**B**) Schematic presentation of a volume profile.

The fundamental principle behind the concept of performing high-pressure mechanistic studies in coordination chemistry in solution is to gain further mechanistic insight on bond formation and bond cleavage reactions, as well as electron transfer reactions, on the basis of partial molar volume changes. Pressure acceleration is coupled to a negative volume of activation, whereas pressure deceleration is coupled to a positive volume of activation, as illustrated in the following two slides (see Scheme 1).

In the case of reversible reactions, volume profiles can be constructed on the basis of the measured activation volumes for the forward and back reactions, or the reaction volume obtainable from the pressure dependence of the overall equilibrium constant K. A few examples of volume profiles are presented in the subsequent sections. 

During the 1960s and 1970s, coordination chemists took the challenge to develop especially high-pressure instrumentation to study fast reactions on the stopped-flow, T-jump, flash-photolysis, and NMR timescale. Up until then, high-pressure instrumentation was available to study slow reactions as a function of hydrostatic pressure up to 2–3 kbar (200–300 MPa) with a half-life longer than 15 min to pressurize the system and allow for temperature and pressure equilibration. In principle, one could use a commercial autoclave with a large Teflon bulge filled with a solution, and withdraw samples from time to time to follow the progress of the reaction as a function of pressure. 

**Figure 1:**
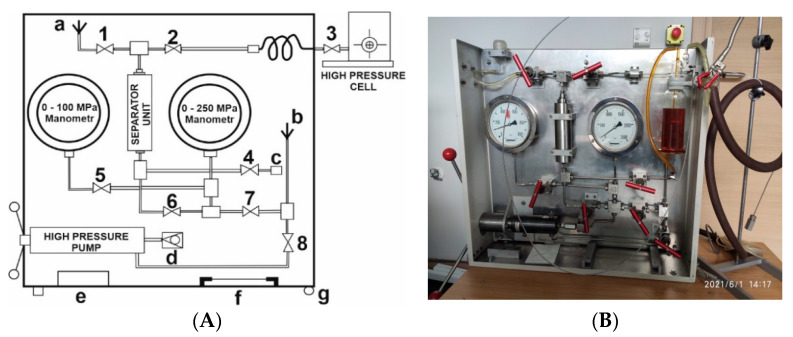
(**A**) Schematic diagram of transportable high-pressure unit complete with high-pressure cell. (a) Pressure medium reservoir, (b) oil reservoir, (c) to vacuum line, (d) one-way valve, (e) holder for pump handles, (f) holder for high-pressure cell, and (g) wheels for easy transportation. Numbers 1–8: high-pressure valves that enable the pressurization of particular sections of the high-pressure unit. (**B**) Picture of transportable suitcase. Reproduced from Reference [18], with the permission of AIP Publishing.

Different types of pressurizing systems exist, consisting of a high-pressure pump, the possibility to change from a hydraulic fluid to another pressurizing liquid (such as water for optical measurements) with the help of a Teflon bulge, pressure gauges, and a series of pressure valves, all built into a compact unit for easy transportation, as shown in Figure 1 [18]. Such transportable units were constructed for use at the Brookhaven National Laboratory, New York, for flash-photolysis and pulse-radiolysis experiments, for pulse-radiolysis experiments at the Hebrew University in Jerusalem, and flash-photolysis experiments at the Jagiellonian University in Krakow, Poland. It enabled the performance of high-pressure kinetic experiments practically all around the world. 

**Figure 2:**
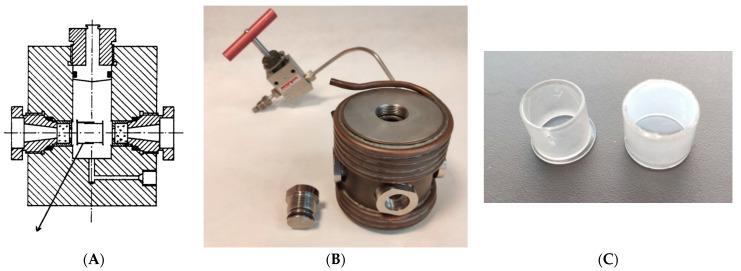
(**A**) Two-window optical high-pressure cell consisting of 1 cm–thick quartz windows and sealed by O- and ∆-rings for pressures up to 200 MPa. Usually the pressure medium is water and is thermostated by an outer copper coil not shown in the diagram. The pill-box consists of two closely fitting quartz cylinders which allow compression of the solution inside the cell. There is a special hole drilled through both cylinders by which the pill-box optical cell can be filled and sealed by turning the two cylinders 180° apart. (**B**) Four-window optical high-pressure cell with a thermostating copper coil. (**C**) Two separated cylinders of the pill-box optical cell that make a gastight fit. Reproduced from Reference [18], with the permission of AIP Publishing.

An optically see-through two-to-four-window high-pressure cell, as shown in Figure 2, can be used to monitor reaction progress as a function of time, temperature, and pressure for slow (dead time ca. 15 min) and fast (flash-photolysis and pulse-radiolysis) reactions in coordination chemistry [18].

Subsequently, a special technique was developed to fill the pill-box optical cell under un-aerobic conditions [19]. In later developments, the same high-pressure cell, but in a four-window version, was used to perform flash-photolysis and pulse-radiolysis experiments as a function of pressure (see further details given below). In further developments, a very compact high-pressure cell was constructed that enabled spectroscopic studies in the UV–Vis–NIR range up to 400 MPa [20]. In this case the pill-box cell could not be employed any longer, but instead round glass or quartz tubes were used to separate the sample solution from the secondary high-pressure fluid (Figure 2C). Depending on the extinction coefficient of the sample to be studied, two different sample tubes with 7.8 mm outer diameter were used: for samples with a high extinction coefficient, a tube with an inner diameter of 1–2 mm was used; for samples with a low extinction coefficient, a tube with an inner diameter of about 5.5 mm was used. In both cases, the tubes were sealed with moving pistons to transmit the pressure, as described in more detail in the original manuscript [20].

**Figure 3:**
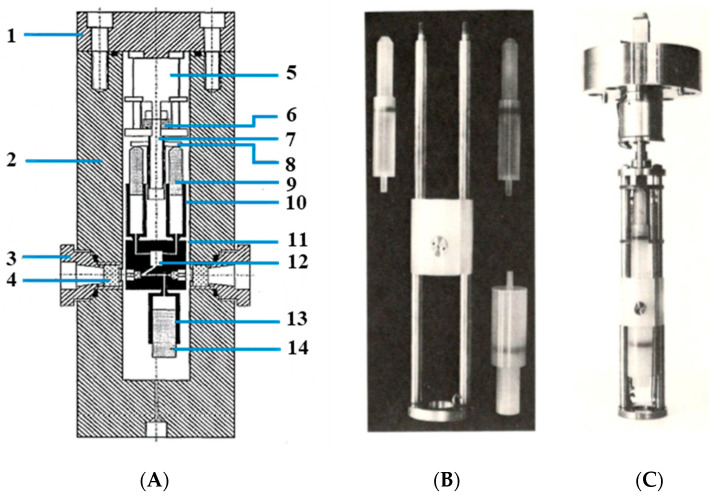
(**A**) Cross-sectional diagram of the high-pressure vessel and stopped-flow apparatus. (1) Pressure vessel lid, (2) pressure vessel, (3) high-pressure window seal assembly, (4) high-pressure sapphire window, (5) step motor, (6) screw-drive mechanism, (7) drive shaft, (8) syringe thrust plate, (9) reactant syringe pistons, (10) reactant syringes, (11) Kel-F cell block, (12) mixing jet, (13) receiver syringe, and (14) receiver-syringe piston. (**B**) High-pressure stopped-flow instrument as constructed by van Eldik and co-workers [21]. Essential components, such as syringes and mixing chamber. (**C**) Complete unit attached to the lid of the pressure cell. Reproduced from Reference [21], with the permission of AIP Publishing.

The limitations in terms of a faster timescale could only be overcome by turning to stopped-flow techniques by which reactants could be mixed rapidly. This required the construction of a complete stopped-flow device within a high-pressure cell. The details of this construction are shown in Figure 3 [21]. This high-pressure stopped-flow unit was designed in such a way that the sample syringes contained ca. 2 mL of solution but enabled more than 20 kinetic traces to be recorded with a single filling of the sample syringes [21]. At the start of the measurements, the receiver syringe was filled with solvent and fully emptied by flushing the optical path in an upward direction. The filled sample syringes were then mounted; at the end of the experiment, the sample syringes were empty, and the receiver syringe was filled with the product mixture. Under favorable conditions, it was possible to complete a whole pressure dependence consisting of four pressures, between 5 and 150 MPa, and five kinetic experiments at each pressure. The dead-time of the stopped-flow unit was determined to be 100 ms from the time the step-motor was triggered to the time complete mixing of the reactants was achieved.

**Figure 4:**
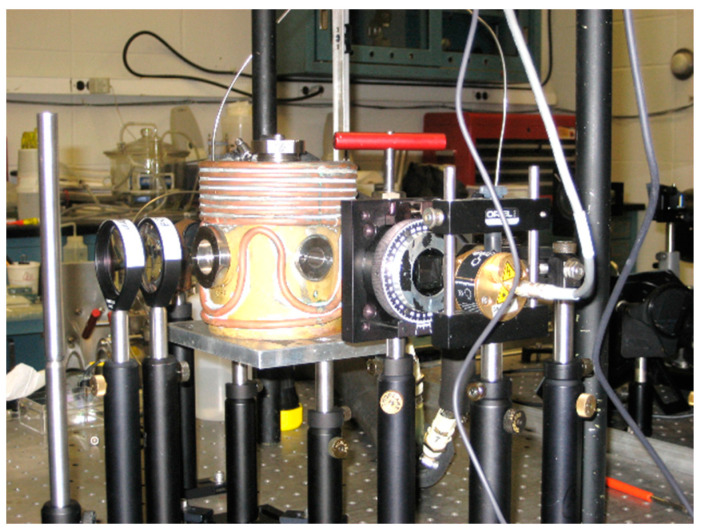
A four-window high-pressure cell built into a pulsed-laser flash photolysis unit with an optical detection system, using the pill-box cell shown in Figure 2, which allows a perpendicular alignment of the laser beam and optical detection system.

The next development was the application of pulsed-laser flash-photolysis. In this case, a four-window high-pressure cell was used in a perpendicular way to flash and trigger the optical detection system after a short time delay in the nanosecond time range [18]. Flash photolysis is a powerful technique to study the reaction mechanism of coordination and organometallic compounds in solution, where the pressure variable adds a further dimension to the mechanistic analysis in terms of volume changes associated with the formation of the transition state. A laser-pulse disturbs the existing equilibrium situation, on which the subsequent recombination reaction allows to follow the kinetics of the studied reaction on a micro- to nanosecond timescale. A typical laser-pulsed flash photolysis setup is shown in Figure 4. 

**Figure 5:**
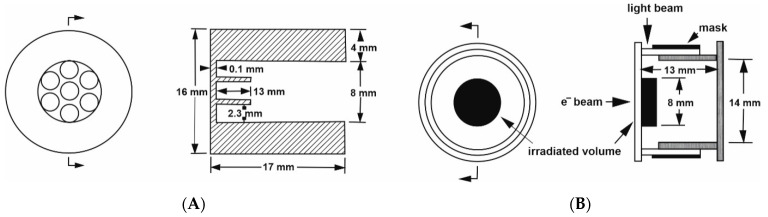
(**A**) Schematic diagram of the electron beam window for high-pressure pulse radiolysis. The electron beam enters from the right side. (**B**) Schematic diagram of the pill-box sample cell, showing the electron beam and light beam paths and the irradiated volume. Reproduced from Reference [22], with the permission of AIP Publishing.

A further development was the challenge to perform pulse-radiolysis experiments at elevated pressure to study the formation of radicals that can undergo ligand substitution and electron transfer reactions. This was not an easy task, since the optical quartz windows used in the standard high-pressure cell did not allow an electron beam to reach the sample within the pill-box cell. Therefore, we had to construct a special steel window that consisted of a single 8 mm hole that was 13.3 mm deep, in combination with seven 2.3 mm holes at 4.3 mm deep, to form a metal grid of 0.7 mm steel that enables the electron beam to enter the high-pressure cell [22]. The construction of the steel window was such that it met the pill-box cell on the flat optical window, allowing for very close contact with the pressure medium. The details of this construction are shown in Figure 5A,B.

**Figure 6:**
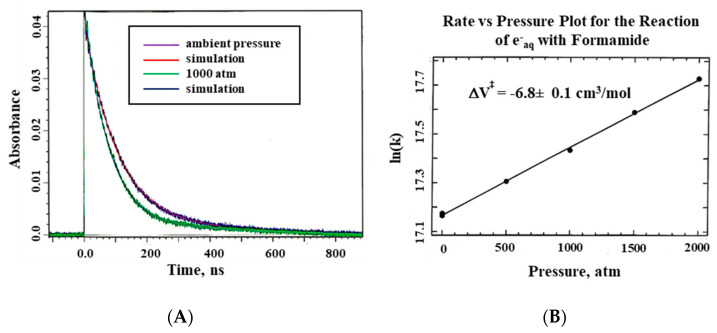
(**A**) Typical kinetic traces to show the acceleration of the reaction at 100 MPa induced by the LEAF facility at BNL, New York. (**B**) Plot of ln*k* versus pressure, where ΔV^‡^ was calculated from the slope of the linear dependence on pressure.

The system described here was used for all pulse radiolysis experiments performed at Brookhaven National Laboratory, New York, using the Laser Electron Accelerator Facility (LEAF), and the Hebrew University in Jerusalem [22]. Typical kinetic traces showing that the electron beam indeed reaches the formamide sample within the pill-box cell are presented in Figure 6 on a nanosecond timescale.

**Figure 7:**
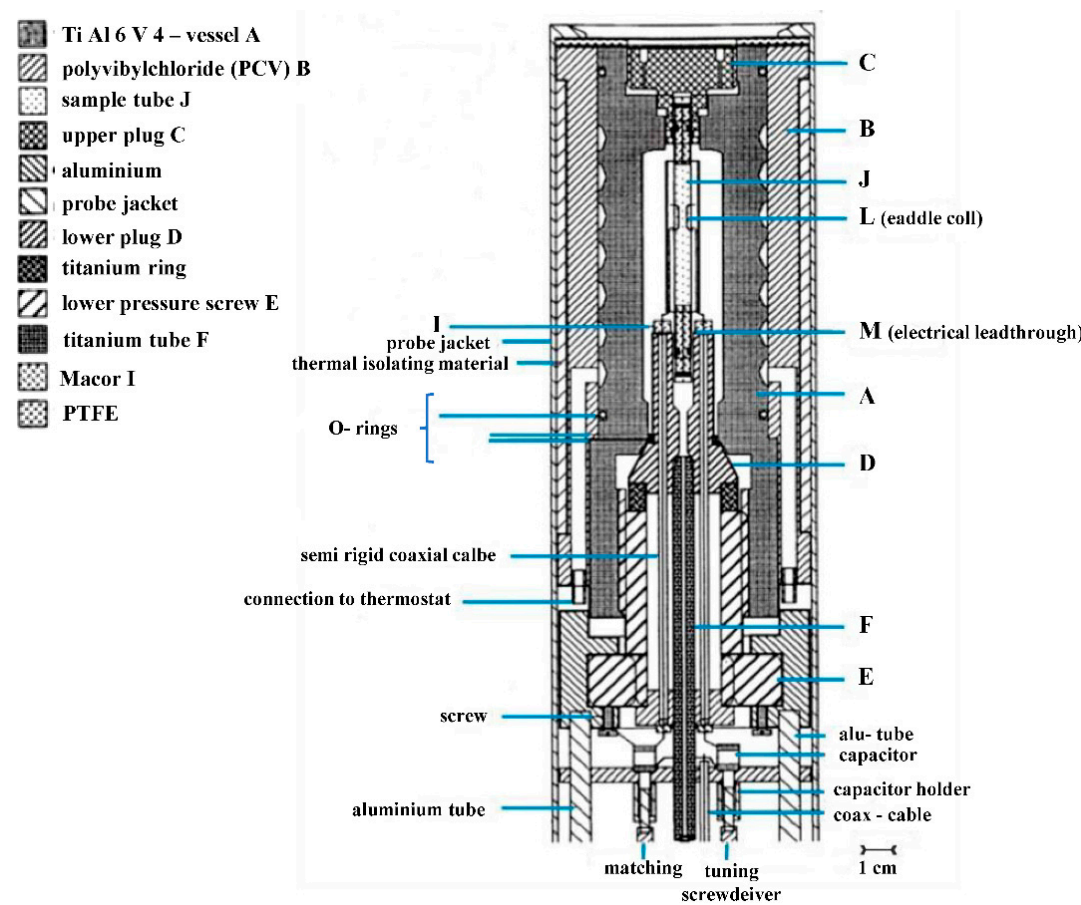
Schematic diagram of the titanium high-pressure NMR probe-head designed for a wide-bore magnet. Reproduced from Reference [23], with the permission of AIP Publishing.

**Figure 8:**
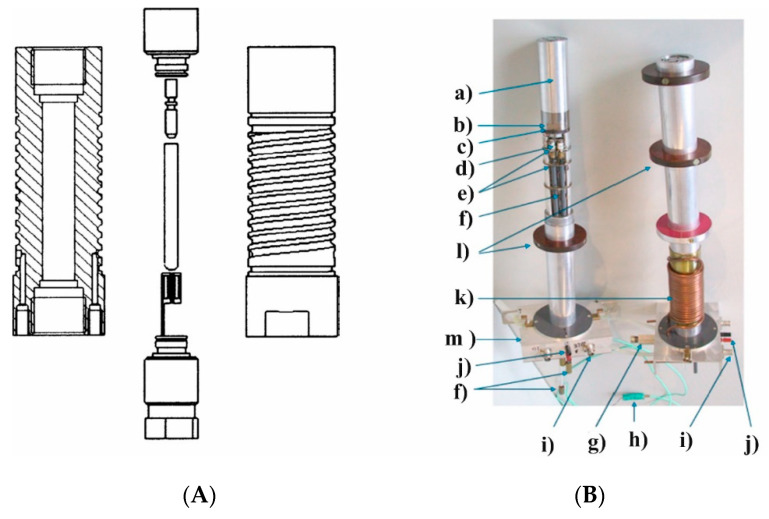
(**A**) Cross-sectional view of the high-pressure autoclave with top and bottom plug, sample tube, macor plug and sample coil. (**B**) Photograph of two narrow bore probe heads: (a) aluminum jacket sealing the double helix used for thermostating, (b) high-pressure vessel, (c) platform carrying the autoclave, (d) capacitors, (e) capacitor platforms, (f) tuning rods, (g) high-pressure connector, (h) thermocouple, (i) BNC connector, (j) Pt-100 connector, (k) copper tubing, and (l) wide-bore adapter. Reproduced from Reference [24], with the permission of AIP Publishing.

Our next challenge was to construct a high-pressure probe for a Bruker 400 MHz NMR machine with a wide bore magnet and a 9 cm cylindrical bore. The wide-bore probe could be tuned for various isotopes, such as ^1^H, ^13^C, ^15^N, ^17^O, and ^31^P, with a pressure range of 200 MPa. The construction of this probe was completed in 1994 (see Figure 7) [23]. Ten years later, we were successful to build a narrow-bore high-pressure probe that could fit a standard magnet with a 3.5 cm cylindrical bore (see Figure 8) [24]. The unique possibility to exchange normal and high-pressure probes from the bottom of the NMR magnet, made it easy to perform high-pressure measurements since everything could be tuned in terms of temperature, frequency and pressure from the bottom of the magnet. The Bruker Company also now has a high-pressure unit available for their range of NMR machines.

**Figure 9:**
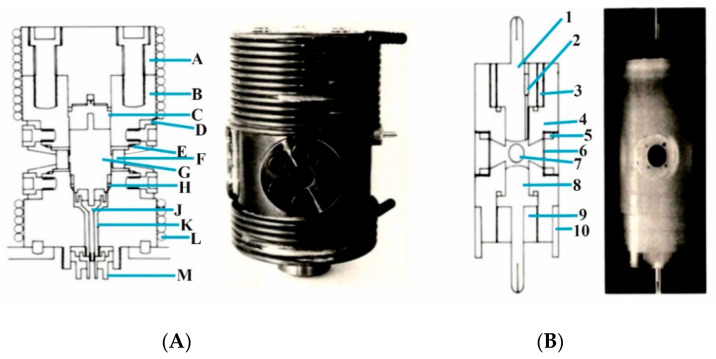
(**A**) High-pressure temperature-jump cell as designed by Doss et al. High-pressure cell: A—vessel lid, B—vessel body, C—steel piston, D—window support, E—∆- and O-rings, G—space for T-jump cell, H—∆- and O-ring, J—steel high-voltage connector, K—insulation material, L—circulation coil, and M—high-voltage plug. (**B**) T-jump cell: 1—upper electrode, 2—deaeration hole, 3—electrode thread support, 4—Kel-F cell body, 5—membrane support, 6—Teflon membrane, 7—optical window, 8—lower electrode, 9—threaded bolt, and 10—support ring. Reproduced from Reference [25], with the permission of AIP Publishing.

Finally, a PhD student at the University of Frankfurt am Main constructed a joule-heating temperature-jump cell that enabled a discharge of 20 kV to “jump” the temperature of the solution by 2–3 °C, followed by the relaxation of the studied equilibrium to the new temperature for pressures up to 200 MPa [25]. The high-pressure cell itself is shown in Figure 9A, whereas the temperature-jump cell is shown in Figure 9B. The latter cell was constructed from Kel-F and had two optical windows for the photometric detection of the absorbance changes as a function of time, and two Teflon membranes by which the pressure was transmitted to the inside of the Kel-F cell. Gold electrodes were used for the rapid discharge of 20 kV. The high-pressure cell was mounted on a standard base plate of a Messanlagen Studiengesellschaft (Göttingen, Germany) T-jump instrument. In conclusion, it can be said that very similar effects are observed with a joule-heating temperature-jump system than with a laser-heated analog when inorganic complex formation processes of the type investigated are studied. 

## 3. NO Binding to Heme Proteins and Model Iron Porphyrin Complexes

Mechanistic studies on the NO binding to iron(II) and iron(III) porphyrin model systems, as well as heme proteins, provided answers to numerous questions related to the influence of the radical character of NO on its reaction mechanism with an iron center, the role of the metal–ligand system, the role of the amino acid residues surrounding the active center, and the protein superstructure. The activation volume parameter (ΔV^‡^), determined in high-pressure stopped-flow or laser-flash photolysis studies, provided information that was particularly important in the process of unraveling the reaction mechanisms of formation and decay of nitrosyl hemoproteins. Ford and co-workers [26] performed systematic hydrostatic pressure (0.1–250 MPa) measurements and determined ΔV^‡^ parameters for the “on” and “off” reactions with ferric, as well as for “on” reactions with ferrous water-soluble porphyrins (Table 1), where TPPS represents the tetrakis(4-sulfonatophenyl)porphinato anion, and TMPS represents the tetrakis(sulfonatomesityl)porphinato anion (Table 1).

**Figure 10:**
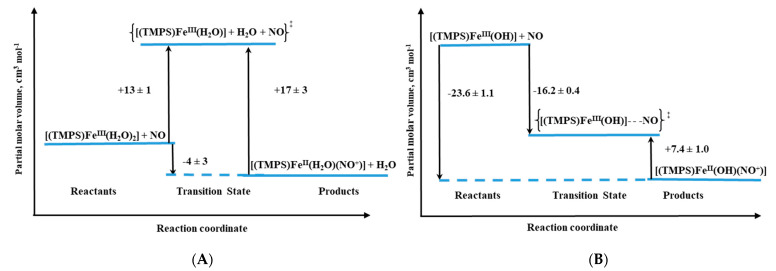
Volume profiles for reversible NO binding to Fe(TMPS) (**A**) (TMPS)Fe^III^(H_2_O)_2_ and (**B**) (TMPS)Fe^III^(OH) under acidic and alkaline conditions, respectively.

Volume profiles that clearly visualize the position of the transition state can be constructed based on the obtained activation volumes. Examples of volume profiles constructed for the ferric TMPS porphyrin are presented in Figure 10 as a function of pH.

For both ferri– and ferro–diaqua–heme models, positive ΔV^‡^_on_ values were determined (Table 1) [26]. Due to the six-coordinate nature of ferri–heme complexes, the large positive ΔV^‡^_on_ values provided support for a dissociatively activated mechanism with the limiting step expressed by Equation (1).

(1) $$(\mathrm{Por}){\mathrm{Fe}}^{\mathrm{III}}(H_{2}O{)}_{2}{}_{\underset{k_{-1}}{\leftarrow }}^{\overset{k_{1}}{\to }}(\mathrm{Por}){\mathrm{Fe}}^{\mathrm{III}}(H_{2}O)+H_{2}O$$

(2) $$(\mathrm{Por}){\mathrm{Fe}}^{\mathrm{III}}(H_{2}O)+\mathrm{NO}{}_{\underset{k_{-2}}{\leftarrow }}^{\overset{k_{2}}{\to }}(\mathrm{Por}){\mathrm{Fe}}^{\mathrm{II}}(H_{2}O)({\mathrm{NO}}^{+})+H_{2}O$$

Further support for this conclusion was provided by high-pressure NMR studies on the water-exchange kinetics of the diaqua ferric porphyrins, performed in the van Eldik group [34], which confirmed that the activation parameters for the nitrosylation reaction of (Por)Fe^III^ are largely defined by a dissociative mechanism. The mentioned studies allowed concluding that the lability of coordinated water plays a crucial role in the dynamics of the reversible NO binding to ferrous porphyrins, whereas the free radical nature of NO has only a minor (if any) influence at all. 

**Figure 11:**
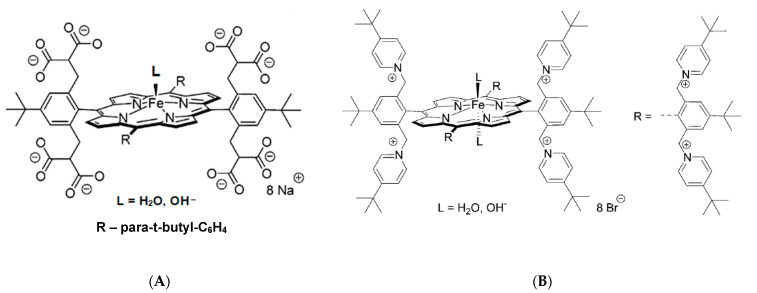
Structure of (**A**) (Por^8+^)Fe^III^ and (**B**) (Por^8−^)Fe^III^. Reprinted with permission from *Inorganic Chemistry*, **2005**, *44*, 7717–7731 Copyright 2005 *American Chemical Society and Inorganic Chemistry*, **2006**, *45*, 1326–1337. Copyright 2006 American Chemical Society [28,29].

This conclusion was further supported by detailed studies with the application of highly negatively charged, (Por^8−^)Fe^III^, and positively charged, (Por^8+^)Fe^III^, water-soluble porphyrins (Figure 11) [28,29]. The determined activation parameters, pointed towards dissociative or interchange dissociative mechanism for NO binding to (Por^8−^)Fe^III^ and interchange dissociative (I_d_) or even interchange (I) for (Por^8+^)Fe^III^ (Table 1). This was reflected by much higher ΔV^‡^_on_ values obtained for the nitrosylation of (Por^8−^)Fe^III^ than for (Por^8+^)Fe^III^, for which substantially smaller ΔV^‡^_on_ values were found. These studies revealed that the nature and charge of the substituents on the porphyrin ring tune the lability of coordinated water by affecting the electron density present on the iron(III) center. In porphyrins with electron-donating substituents, breakage of the Fe-H_2_O bond is facilitated, whereas an opposite effect (bond stabilization) is a consequence of electron-withdrawing substituents. Such properties result in the observed change in the mechanism of NO binding from predominantly dissociative for the (Por^8−^)Fe^III^ to interchange for (Por^8+^)Fe^III^ [28,29].

The revealed mechanism made it easier to explain the NO reactivity towards iron(II) porphyrins, for which the rate constants were determined to be about three orders of magnitude larger than for its ferric analogues (Table 1). It became clear that due to the five-coordinate nature of high-spin iron(II) porphyrins, no limitations related to ligand substitution occurred. Small, positive ΔV^‡^_on_ values determined for NO binding to iron(II) porphyrins allowed to classify the mechanism as diffusion-limited. In such a mechanism, an encounter complex [(Por)Fe^II^||(NO)] creation before metal–ligand bond formation is the rate-limiting step ($k_{a}\gg k_{-D}$), thus ΔV^‡^_on_ = ΔV^‡^_D_ (activation volume for diffusion) (3) [26].

(3) $$(\mathrm{Por}){\mathrm{Fe}}^{\mathrm{II}}+{\mathrm{NO}}_{\underset{k_{-D}}{\leftarrow }}^{\overset{k_{D}}{\to }}\left[(\mathrm{Por}){\mathrm{Fe}}^{\mathrm{II}}||(\mathrm{NO})]\overset{k_{a}}{\to }(\mathrm{Por}){\mathrm{Fe}}^{\mathrm{II}}(\mathrm{NO}\right)$$

This is in agreement with the fact that positive activation volumes for diffusion in various solvents are a result of the viscosity increase with increasing pressure [35]. The dynamics of NO binding to Fe^II^ porphyrins are different than for CO binding. Several orders of magnitude lower second-order rate constants for CO coordination, together with the negative ΔV^‡^_on_, provide confirmation that in this case, an activation-limited mechanism exists ($k_{-D}\gg k_{a}$). In the activation-limited mechanism (ΔV^‡^_on_ = ΔV^‡^_a_), the negative ΔV^‡^_on_ value is determined by Fe^II^-L bond formation and concomitant change in spin state.

It was surprising to observe similar NO binding dynamics for pentacoordinate ferric-monohydroxo TMPS [27]. In this case, much lower reaction rates, concomitant with negative ΔV^‡^_on_ allowed to conclude that the reactivity pattern is not always controlled by the lability of the iron(III) center (as would be expected in the case of the diffusion-limited reactions), but can be determined by the spin density reorganization and accompanying structural changes [27]. Comparison of typical volume profiles determined for six-coordinate diaqua vs. five-coordinate monohydroxo iron(III) porphyrins are presented in Figure 10.

Comparison of the results obtained for the model porphyrins with the ones for ferriheme proteins was crucial to reveal the role of an amino acid chain surrounding the active center: polarity of the protein pocket, hydrogen-bonding interactions, prosthetic group solvent-accessibility, etc. The first high-pressure studies concerning reversible NO binding by heme protein were performed for met-myoglobin (metMb). A successful collaboration of three research groups (G. Stochel, R. van Eldik and P. Ford) resulted in a joint publication reporting high-pressure effects on the nitrosylation reaction rates [30]. The semi-independent results were performed by using two techniques: laser flash-photolysis and stopped-flow. The application of these two fundamentally different techniques resulted in the determination of a set of activation parameters being in good agreement and providing insight into the understanding of the nitrosylation mechanism of met-Mb. The large and positive ΔV^‡^_on_ (Table 1) and ΔS^‡^_on_ values indicated a limiting dissociative mechanism, controlled by the lability of a water molecule, similar to the reactivity pattern observed for water-soluble Por systems. Attention was drawn to the fact that the value of ΔV^‡^_on_ for met-Mb nitrosylation is significantly larger than the corresponding value for ferric-porphyrin model systems. This observation was ascribed to the structural reorganization occurring in the protein pocket upon coordinated water release [30].

**Figure 12:**
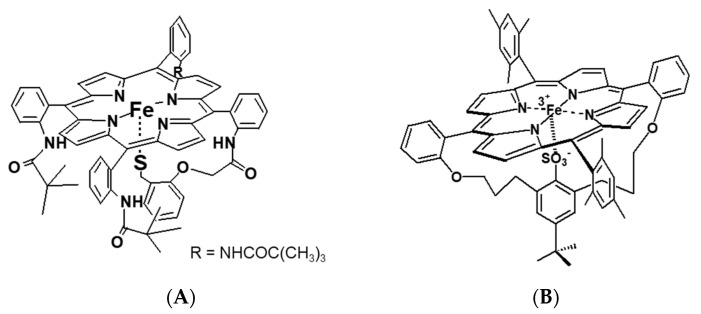
(**A**) Synthetic heme–thiolate complex, SR complex, (**B**) model of P450 bearing R-SO_3_^−^ as an axial ligand (R-SO_3_^−^). Reprinted with permission from *Journal of the American Chemical Society* **2005**, *127*, 5360–5375 Copyright 2005 American Chemical Society and *Journal of the American Chemical Society* **2006**, *128*, 13611–13624 Copyright 2006 American Chemical Society [32,33].

Besides myoglobin, particular attention was also devoted to the reactivity of cytochrome P450 towards small molecules (O_2_, NO, and CO). Cytochromes P450, due to their important biological functions, untypical active site with cysteine thiolate ligation to the iron center, and spin-state equilibria of the resting state, are subjects of intensive research. Detailed mechanistic studies on P450 nitrosylation played a crucial role in understanding the biological function principle of this protein. High-pressure studies on the reversible NO binding by two forms of P450, namely substrate-free (six-coordinate, low-spin complex with water coordinated trans to Cys) and camphor-bound (five-coordinate, high-spin complex), as well as the heme–thiolate porphyrin model (Figure 12A), also contributed to this aspect [31,32].

The activation volumes determined for NO binding to these two P450 forms revealed different nitrosylation mechanisms [31]. For substrate-free cytochrome P450 a mechanistic scenario similar to the one for metMb was observed, which is reflected in positive values of ΔV^‡^_on_ and ΔS^‡^_on_ (Table 1). The positive ΔV^‡^_on_ corresponds to a typical limiting dissociative ligand-substitution mechanism, in which the dissociation of coordinated water limits the NO binding rate. Significantly larger ΔV^‡^_on_ = +28 cm^3^/mol in comparison to metMb represents a much higher structural rearrangement in the protein pocket of substrate-free P450, as well as a spin-state change on going from the low-spin six-coordinate complex to high-spin five-coordinate transition-state intermediate [31]. The much smaller positive ΔV^‡^_on_ = 6 cm^3^/mol was determined for NO binding to synthetic heme–thiolate six-coordinate complex (SR) in methanol [32], a model of P450 cytochrome, allowing us to estimate the participation of factors other than Fe^III^-H_2_O bond breakage (spin-state change and reorganization in the protein pocket). 

For camphor-bound P450 (P450(camph)), a completely different mechanistic pathway was observed that is expressed by negative values for both activation volume (Table 1) and entropy determined for NO binding. Substantially negative ΔV^‡^_on_ is consistent with the five-coordinate nature of P450(camph) form, for which the formation of [(Por)Fe^III^||(NO)] intermediate complex before NO bonding is expected [31].

**Figure 13:**
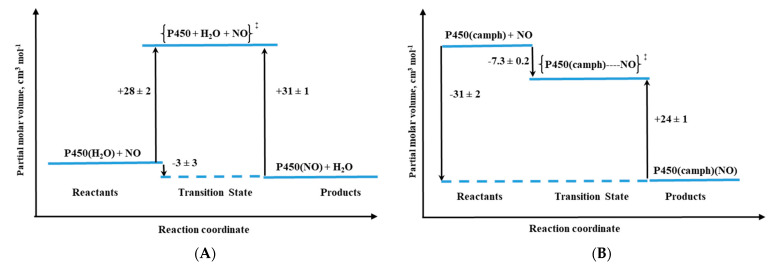
Volume profiles for reversible NO binding to (**A**) substrate-free (P450) and (**B**) camphor bound (P450(camph)) cytochrome.

**Figure 14:**
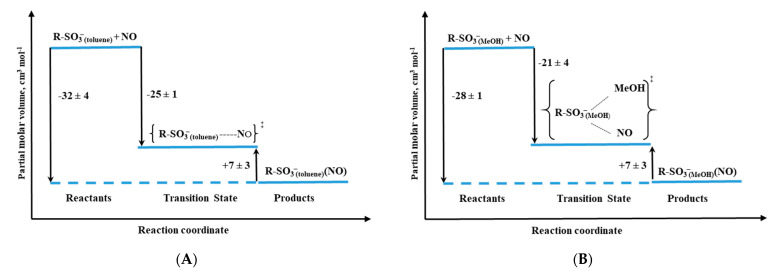
Volume profiles for reversible NO binding to R-SO_3_^−^ model in (**A**) toluene (R-SO_3_^−^_(__toluene)_) and (**B**) in methanol (RSO_3_^−^_(MeOH)_).

Other studied valuable cytochrome P450 models were iron–porphyrin complexes, in which the thiolate group was substituted by a R-SO_3_^−^ ligand (Figure 12B). Depending on the solvent applied in the study, non-coordinating toluene vs. coordinating methanol, the RSO_3_^−^ complex exists as five-coordinate or six-coordinate species, respectively [33]. Based on the activation parameters, it was clear that both the R-SO_3_^−^ complex in toluene (R-SO_3_^−^ _(toluene)_) and five-coordinate P450(camph) follow the same mechanism, dominated by Fe-NO bond formation. However, a comparison of the activation volume profiles indicated differences in the transition states arising from dissimilar contributions from bond formation and spin state change (compare Figure 13B and Figure 14A). Surprisingly, the reactivity profile of the six-coordinate R-SO_3_^−^complex in methanol (R-SO_3_^−^_(MeOH)_) differs from the six-coordinate substrate-free P450. High-pressure studies on the NO binding to the R-SO_3_^−^_(MeOH)_ complex revealed a significantly negative ΔV^‡^_on_ value, indicating a volume collapse ongoing from the substrate to the transition state. An associative interchange mechanism determined for the R-SO_3_^−^_(MeOH)_ complex was related to the high spin nature of the complex, which is believed to govern the reactivity mechanism. This contrasts the low-spin six-coordinate P450 case, as the dissociative mechanism controls nitrosylation in such a system. The opposite effect is nicely illustrated by the volume profiles reported in Figure 13A and Figure 14B.

As was described above, activation volumes obtained from high-pressure kinetic studies for reversible NO binding to various iron porphyrin model systems, as well as heme proteins, provide crucial information that leads to conclusions regarding the molecular mechanisms that govern the nitrosylation of heme centers. Volume profiles constructed based on the obtained data nicely present the differences in the position of transition states and allow graphically expressing the influence of various factors affecting it. The studies cited above demonstrate the invaluable impact of ΔV^‡^ in revealing the influence of the electronic nature of the porphyrin, the axial ligands present, the type of solvent (coordinating vs. non-coordinating), pH (influencing ionization state of protic groups), and amino acid chain that surrounds the active site on the ligand substitution behavior. Activation volumes are also a significant help in the identification of the relevant factors tuning the biological function of specific proteins.

## 4. Reactions of NO with Model Fe^II^ Complexes

In the literature, we can find examples of the application of high-pressure kinetic methods in mechanistic studies of the reactions of nitric oxide (NO) with iron(II) complexes, e.g., polyaminocarboxylates, cyanoferrates [36,37], or others [38,39]. The importance of mechanistic studies on such complexes is the comparison with water-exchange reactions that were clearly shown to control the nitrosylation process [37,40,41,42,43,44]. In this section, we discuss a few important systems in which the high-pressure measurements helped to understand the nature of the underlying reaction mechanism.

An interesting example of the application of high-pressure methods to improve our understanding of the underlying mechanism is the reaction known to every high school chemistry student, the so-called “brown-ring test” for nitrate [44]. The mentioned reaction is used as a “chemical test” that helps to demonstrate the presence of nitrate or nitrite ions in an aqueous solution. The test consists of the reduction of nitrate and nitrite to NO by an excess of Fe(II), which in the presence of concentrated sulfuric acid forms a characteristic brown-ring. Although the reaction is known for more than 100 years, the details of its mechanism were investigated relatively recently [45]. The process involves the formation of a characteristic green-brown colored ring at the junction of the two solutions layers. This brown-green color is provided by the formation of a Fe-NO complex in the reaction between [Fe(H_2_O)_6_]^2+^ and NO (see Reaction (4)):

(4) $${\left[{\mathrm{Fe}}^{\mathrm{II}}{\left(H_{2}O\right)}_{6}\right]}^{2+}+\mathrm{NO}⇄{\left[{\mathrm{Fe}}^{\mathrm{II}}{\left(H_{2}O\right)}_{5}\left(\mathrm{NO}\right)\right]}^{2+}+H_{2}O$$

During the reaction, one water molecule in [Fe(H_2_O)_6_]^2+^ is displaced rapidly by the NO molecule (*k*_on_ = 1.42 × 10^6^ M^−1^ s^−1^). Detailed studies [44] showed that the product is formally stabilized as the Fe^III^-NO^−^ complex in which electron density is shifted from the metal center to the ligand that results in the reduction of NO to NO^−^ and formation of the iron(III)-nitrosyl complex. The back reaction involves the formation of [Fe(H_2_O)_6_]^2+^ and release of NO (*k*_off_ = 3240 ± 750 s^−1^) (see reaction 4).

**Figure 15:**
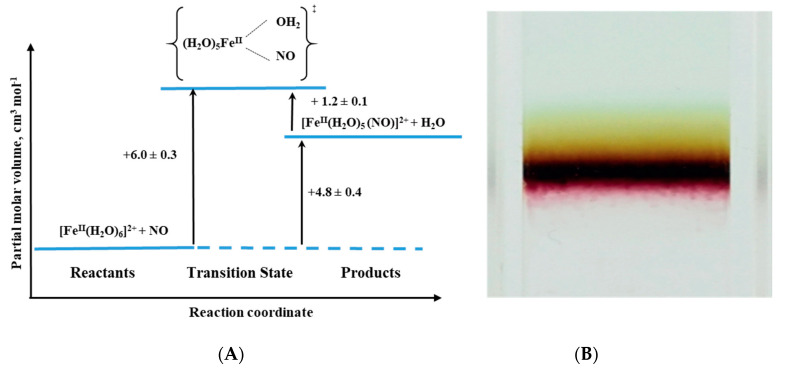
(**A**) Volume profile for the reaction of [Fe(H_2_O)_6_]^2+^ with NO. (**B**) A picture of the brown-ring formed during the test for nitrate or nitrite [45]. Copyright Wiley-VCH GmbH. Reproduced with permission.

The effect of pressure on the described reaction was studied using the high-pressure flash-photolysis technique in the pressure range 0.1–170 MPa (20 °C, pH = 5.0, 0.2 M acetate buffer) resulting in small positive volumes of activation (Table 2): +6.1 ± 0.2 cm^3^ mol^−1^ and +1.3 ± 0.2 cm^3^ mol^−1^ for the “on” and “off” reactions, respectively. The activation parameters obtained from the applied measurements suggest that in both processes ligand substitution follows a dissociative interchange (I_d_) process [44]. Thus, it can be concluded that the forward reaction involves partial Fe-H_2_O bond breakage, prior to NO binding, whereas in the back reaction the Fe-NO bond partially dissociates and is followed by the coordination of H_2_O. This is shown in the volume profile for the reaction presented in Figure 15.

Mechanistic studies on the water-exchange reaction in [Fe(H_2_O)_6_]^2+^ were performed using HP ^17^O-NMR techniques as described in Section 2 of this review. The water-exchange reaction has a rate constant of 4.4 × 10^6^ s^−1^ at 20 °C and the volume of activation for this reaction was reported to be +3.8 ± 0.2 cm^3^ mol^−1^, which suggests that it also follows a dissociative interchange (I_d_) mechanism [40]. This means that both the nitrosylation and water-exchange reactions occur according to the same I_d_ mechanism for which the water-exchange process controls both the rate and the nature of the NO binding mechanism. In a recent publication by Klüfers and Monsch [45], the crystal structure of [Fe(H_2_O)_5_(NO)]^2+^ was resolved, and the Fe-N-O had a bond angle of 168°. This reopened the discussion on the nature of the Fe-NO complex, whether it is Fe(I)-NO^+^, Fe(II)-NO^•^, or Fe(III)-NO^−^ [45]. 

**Figure 16:**
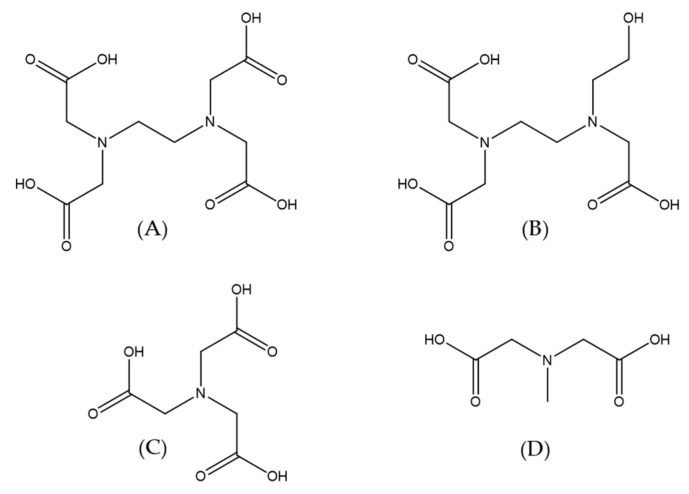
Structures of the ligands: (**A**) edta (ethylenediaminetetraacetate), (**B**) hedtra (hydroxyethylenediaminetriacetate), (**C**) nta (nitrilotriacetate), and (**D**) mida (methyliminodiacetate).

Another interesting system in which the application of high-pressure methods was invaluable for the investigation of reaction mechanisms is the reaction of NO with a group of polyaminocarboxylate complexes of Fe^II^ (edta, hedtra, nta, and mida; see Figure 16) [37]. The performed studies, including an investigation of the coordinated water-exchange process [42,46], allowed us not only to recognize the sequence of the reaction but also gave us crucial answers for questions concerning the mechanistic nature of the studied complexes. In general, polyaminocarboxylate complexes of iron(II) are known to be highly effective NO scavengers due to their very efficient coordination of NO [47,48]. Since it is known that polyaminocarboxylates possess the desired pharmacokinetic property, their iron(II) complexes were considered as reagents that control the NO level in the bloodstream [49,50]. On the other hand, they have been used for the removal of NO from exhaust gasses, since they can enhance the solubility of NO in aqueous solutions.

The studied complexes are seven-coordinate species with one water molecule that is bound to the iron center (for edta, hedtra, and mida) or six-coordinate species with two coordinated water molecules (for nta). It is also worth mentioning that the polyaminocarboxylate complexes of Fe(II) are extremely oxygen-sensitive; in the presence of O_2_ they form Fe(III) species that do not interact with NO at all. The reaction between the group of complexes ([Fe^II^(L)(H_2_O)_n_] and NO, can be expressed by Reaction (5) (see below) and lead to the formation of stable adducts of Fe^II^(L)(NO), where L = edta, hedtra, nta, and mida [37].

(5) $$[{\mathrm{Fe}}^{\mathrm{II}}\left(L\right){\left(H_{2}O\right)}_{n}]+\mathrm{NO}⇄[{\mathrm{Fe}}^{\mathrm{II}}\left(L\right)\left(H_{2}O{)}_{n-1}\left(\mathrm{NO}\right)\right]+H_{2}O$$

Interestingly, similar to that described in the previous example, the nitrosyl products are stabilized as Fe^III^-NO^−^ complexes, due to a shift in charge density from the metal center to the NO ligand. It is worth noting that the observed nitrosylation reactions are very efficient with rate constants that vary from 1.6 × 10^6^ M^−1^ s^−1^ for the (mida)_2_ complex to even 2.4 × 10^8^ M^−1^ s^−1^ for the Fe^II^(edta) complex! The rate constants for the nitrosylation reaction of the complexes increases in following order: edta > hedtra > nta > mida > (mida)_2_. The back reactions leading to NO release and formation of [Fe^II^(L)(H_2_O)_n_] complexes (see reaction 5) are within the range from 0.11 to 3.2 × 10^3^ s^−1^ [37].

For this group of compounds, the high-pressure stopped-flow technique was applied to study the pressure effect and allowed to obtain the results listed in Table 2. What is worth to underline for all complexes except for nta, the activation volumes reached small positive values that suggest the dissociative interchange (I_d_) mechanism for the nitrosylation reaction. Thus, the most probable sequence during the reaction is again the partial Fe-H_2_O bond breakage, followed by NO binding as was also described above for the [Fe(H_2_O)_6_]^2+^ complex [37].

**Figure 17:**
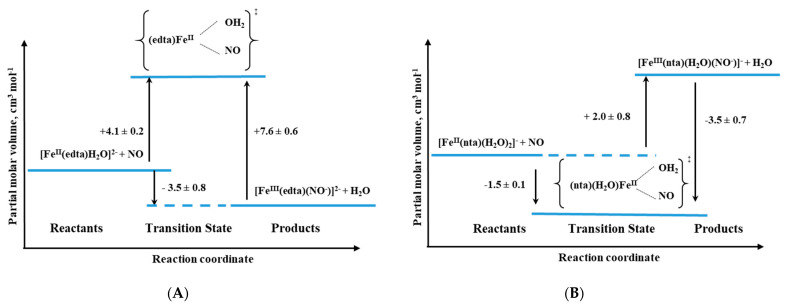
Volume profiles for the reactions of (**A**) seven-coordinate [Fe^II^(edta)(H_2_O)]^2+^ and (**B**) six-coordinate [Fe^II^(nta)(H_2_O)_2_]^2+^ complexes with NO [37].

Only in the case of the nta complex, negative activation volumes were found, indicating an associative interchange (I_a_) mechanism for the nitrosylation reaction and suggesting that the reaction involves partial Fe-NO bond formation prior to the release of H_2_O. This perfectly agrees with the six-coordinate nature of the nta complex compared to the seven-coordinate nature of the other complexes as shown in Figure 17 and the results in Table 2.

Additional ^17^O-NMR studies of the water-exchange reaction for this group of complexes demonstrated that the water-exchange process controls the substitution of NO by Fe(II) polyaminocarboxylate complexes [37,42,43,46]. On the other hand, it was shown that the chelate ligand controls the rate of the water-exchange process, but also has an influence on the nature of the underlying mechanism [42,43,46].

The difference in the nitrosylation mechanism for the nta complex was accounted for as a result of its six-coordinate nature in solution and 18 valence electron character, compared to the other complexes that are seven-coordinate and possess 20 valence electron character. The observed volume changes are related to exchanging H_2_O for the NO ligand in the complexes (seven- or six-coordinate) and likely changes in the formal oxidation state of the metal center and NO [37].

It is noteworthy to mention that similar activation volume values were found for the forward and backward reactions, i.e., positive activation volumes were found for edta, hedtra, mida, and (mida)_2_ complexes, and slightly negative values for the nta complex (Table 2). These values clearly indicate that the backward reactions for edta, hedtra, mida and (mida)_2_ complexes also follow a dissociative interchange (I_d_) mechanism. The Fe^II^(nta)(NO) system is an exception with an I_a_ mechanism for both the “on” and “off” reactions [37].

The final example we would like to discuss concerns the formation and dissociation reactions of [Fe^II^(CN)_5_(NO)]^3−^, the complex that is formed during the interaction between NO and [Fe^II^(CN)_5_(H_2_O)]^3−^ (see Reaction (6)) [39]. Especially, an interesting aspect of the system seems to be the characterization of the NO release reaction, since the similar iron(III) complex is referred to as nitroprusside ([Fe(CN)_5_(NO)]^2−^), which for decades is well-known as NO-releasing agent in biological medium and is used for clinical purposes [38].

(6) $${\left[{\mathrm{Fe}}^{\mathrm{II}}{\left(\mathrm{CN}\right)}_{5}\left(H_{2}O\right)\right]}^{3-}+\mathrm{NO}⇄{\left[{\mathrm{Fe}}^{\mathrm{II}}{\left(\mathrm{CN}\right)}_{5}\left(\mathrm{NO}\right)\right]}^{3-}+H_{2}O$$

The process of [Fe(CN)_5_(NO)]^3−^ formation was found to be quite efficient with *k*_on_ = 250 ± 10 M^−1^ s^−1^ (25.4 °C, *I* = 0.1 M, pH 7.0), and the back reaction expressed by rate constant *k*_off_ = (1.58 ± 0.06) × 10^−5^ s^−1^ (25.0 °C, *I* = 0.1 M, pH 10.2) [39].

**Figure 18:**
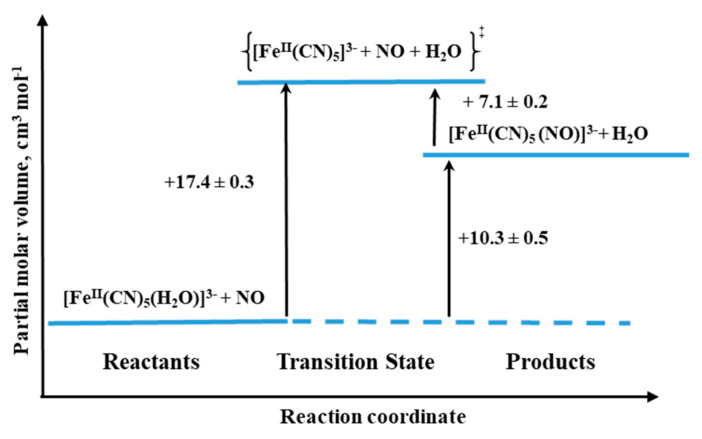
Volume profile for the reversible coordination of NO to [Fe^II^(CN)_5_H_2_O]^3−^ according to Reaction (6) [39].

The activation volumes for both forward and backward reactions were found to be positive: +17.4 ± 0.3 cm^3^ mol^−1^ and +7.1 ± 0.2 cm^3^ mol^−1^ for “on” and “off” reactions, respectively. In the case of the forward reaction, the activation volume value suggests the operation of a limiting dissociative mechanism (D). Thus the rate-controlling process is the dissociation of coordinated H_2_O to form a five-coordinate complex, followed by a fast reaction with NO. For the back reaction, the dissociative mechanism (D) is also favored, which means the reaction involves Fe-NO bond cleavage prior to the coordination of H_2_O (see Figure 18). However, it should be mentioned that NO release could be due to an alternative decomposition route that involves the decay of a tetracyanonitrosyl complex [39].

Finally, it must be mentioned that several studies in which the water-exchange process for a variety of iron(II) and iron(III) complexes, can be found in the literature [40,41,42,43,46]. In these studies, high-pressure methods were employed to assist the understanding of the chemistry of the complexes, the nature of their interaction, the influence of the metal center [42], and the effect of the chelate ligand [41,43] on the water-exchange process.

## 5. NO Binding to Cobalamin and Cobalt Porphyrins

Biological aspects of nitric oxide interactions with metal ions are primarily associated with the activation of metalloenzymes (such as guanylyl cyclase) or their inhibition (observed for cytochrome oxidase or catalase). Interest regarding the reaction mechanisms involving NO has been mostly focused on heme iron complexes and their model systems. Slightly less effort has so far been devoted to cobalt complexes, which have attracted attention due to the known biological effects of the interaction between NO and nitrile hydratase, an enzyme that contains ions of both the aforementioned metals in its active center [51,52]. Studies of cobalt complexes nitrosylation were, therefore, often carried out referring to the better-known iron-based systems. Elucidation of the differences between the reactivity of the two metals towards NO was performed using model porphyrin complexes [53,54]. An expected conclusion from the early studies of this type was the dependence of the reaction course on the oxidation state of the metal and the conditions, pH in particular. Findings by Laverman and Ford, who treated Co^II^(TPPS) to some extent as a reference for iron porphyrin complexes, showed closer similarity between Co(II) and Fe(II) complexes than between Fe(II) and Fe(III) in terms of the reactivity towards NO. The reactions were studied at pH = 7, using high-pressure techniques. The obtained ΔV^#^ values indicate that the reactions outlined in (7)

(7) $$M\left(\mathrm{Por}\right)+\mathrm{NO}\begin{array}{c} k_{\mathrm{on}}\\ ⇄\\ k_{\mathrm{off}} \end{array}M\left(\mathrm{Por}\right)\left(\mathrm{NO}\right)$$

follow D and I_D_ mechanisms for ligand exchange on M(III) and M(II), respectively [26]. In a similar context, Roncaroli et al. reported comprehensive studies on model Co(III) porphyrins [55]. In contrast to M^II^(Por)(NO^+^) species previously postulated as a reaction product of the reactions involving Fe(III) porphyrins, indisputable spectroscopic evidence clearly shows that M^III^(Por)(NO^−^) is formed in the case of Co(III) reactions with NO according to the following reaction sequence:

(8) $${\mathrm{Co}}^{\mathrm{III}}\left(\mathrm{Por}\right)+2\mathrm{NO}\to {\mathrm{Co}}^{\mathrm{III}}\left(\mathrm{Por}\right)\left({\mathrm{NO}}^{-}\right)+{\mathrm{NO}}_{2}^{-}$$

It was observed that the details of the reaction mechanism depended on the reaction medium. To provide in-depth insight into the factors contributing to these differences, the reaction course was followed over a wide range of pH. As the pH was lowered below 3, the characteristics of the kinetic traces changed from biphasic to monophasic, suggesting a single reaction step. Based on the kinetic data, it was concluded that product formation under strongly acidic conditions is preceded by a rate-limiting step of water exchange to form [Co^III^(Por)(NO)(H_2_O)]. The latter species immediately reacts with the second NO molecule. The resulting [Co^III^(Por)(NO)_2_] intermediate is instantly converted to [Co^III^(NO^−^)] by an inner-sphere electron transfer. Reasons for the greater complexity of the mechanism carried out under milder conditions (pH > 3) were perceived in the possible participation of nitrite impurities that undergo protonation in more acidic solutions. The competition of NO_2_^−^ and NO in the formation of the first intermediate justifies the more complex form of the rate law:

(9) $$k_{\mathrm{obs}}=k_{\mathrm{NO}}\left[\mathrm{NO}\right]+k_{{\mathrm{NO}}_{2}^{-}}\left[{\mathrm{NO}}_{2}^{-}\right]$$

In contrast to iron porphyrins, which show relatively high stability of a [Fe^II^(Por)(NO^+^)] intermediate, its cobalt analog, namely [Co^III^(Por)(NO)], is highly prone to further transformations. The formation of a subsequent intermediate, [Co(Por)(NO_2_^−^)(NO)], which slowly decomposes to the final product, was found based on the determined rate constant dependencies on both NO and NO_2_^−^ concentrations, supported by a positive ΔV^‡^ value of +13 cm^3^/mol for Co(TPPS)(H_2_O) at pH = 1. Thus, the mechanism of reductive nitrosylation of cobalt porphyrins differs significantly from the mechanisms of analogous reactions involving iron complexes.

In the human body, cobalt(III) complexes are represented mainly by cofactors of vitamin B_12_. Despite the common macrocyclic framework with porphyrins, the corrin system exerts a peculiar influence on the properties of the metal center, which is of interest, among others, because of the potential activation of the small molecules. Therefore, along with porphyrins, cobalamins (Cbl) represent an attractive and biologically important object of study, for which the application of high-pressure kinetic techniques has contributed to the elucidation of the NO reactions’ mechanisms. Among the naturally occurring vitamin B_12_ derivatives, aquacobalamin (Cbl(H_2_O)) has attracted particular attention because of its substantial ability to substitute the water molecule. Of all the six-coordinated Co(III)-based forms, only one gave rise to a discussion of potential NO binding under physiological conditions [56,57]. However, this hypothesis was negatively verified, and the observed spectroscopic features were attributed solely to the presence of nitrite impurities [58,59]. Under more acidic conditions, Cbl(H_2_O) reacts with NO to give a nitrosyl derivative [60].

Surprisingly, the final product is Cbl^II^(NO), or, given the actual electron density distribution, Cbl^III^(NO^−^), indicating that reductive nitrosylation has occurred. Comparison with previously described porphyrins revealed several similarities but also some differences in the details of these reaction mechanisms. Their convergence is manifested, among others, by the biphasic nature of the reaction occurring in the presence of HNO_2_. The product of the first step, Cbl(NO_2_^−^), is formed at a rate virtually independent of NO concentration and very similar to that observed in the presence of pure HNO_2_ [59]. This finding suggests to rule out the possibility of a direct reaction of Cbl(H_2_O) with NO, regardless of the conditions. At a pH < 4, the nitrite mediated reaction proceeds to form the final product, but the determined concentration dependencies, with respect to both NO and NO_2_, gave rather unexpected courses. While the reaction rate decreases with increasing NO concentration, it increases with the square of [HNO_2_]. Moreover, the reaction yield drops with increasing concentrations of both species. This clearly indicates that the [HNO_2_]-dependent reverse reaction controls the rate of formation of the final product. Hence, the overall course of the reductive nitrosylation of Cbl(H_2_O) is given by the following equation:

(10) $$\mathrm{Cbl}\left({\mathrm{NO}}_{2}^{-}\right)+2\mathrm{NO}+H_{2}O⇄\mathrm{Cbl}\left({\mathrm{NO}}^{-}\right)+2{\mathrm{HNO}}_{2}$$

**Figure 19:**
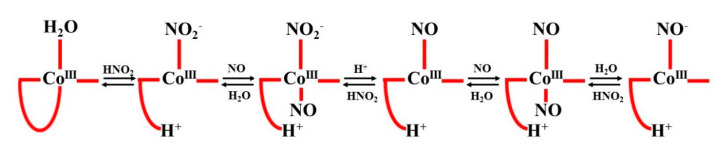
Reaction scheme for the reductive nitrosylation of Cbl(H_2_O) at low pH (according to [17]).

The role of nitrite under acidic conditions was clarified by considering the properties of the dihydrobenzimidazole group (DMBI), which, in the natural “base on” form, coordinates to the cobalt ion on the opposite side of water in Cbl(H_2_O). The substitution of H_2_O for NO_2_^−^ has been shown to markedly shift the p*K_a_* value of DMBI, which, in very acidic solutions, allows dechelation (“base off” formation) and NO binding. The opposite sequence of events cannot be realized. The detailed mechanism of the multi-step reaction of Cbl(H_2_O) with NO in the presence of HNO_2_ at low pH is shown in the following scheme (see Figure 19):

The instability of the Co^III^-NO species, demonstrated previously for Co(III) porphyrins, accounts for the practical inability of Cbl(H_2_O) to react directly with NO. Thus, the mechanism of reductive nitrosylation of Cbl(H_2_O) is significantly different from that of an analogous reaction involving Fe(III) porphyrins.

While the issue of the potential interaction of Cbl(H_2_O) with NO has remained controversial for many years, such a reaction with Cbl(II) is virtually indisputable. It is supported by unequivocal evidence provided by a variety of spectroscopic techniques (UV–Vis, ^1^H-, ^15^N-, and ^31^P NMR). The results of kinetic investigations indicate a very high equilibrium constant (10^8^ M^−1^ at pH = 7.0) for the following reaction:

(11) Cbl(II)+NO⇄Cbl(NO)

**Figure 20:**
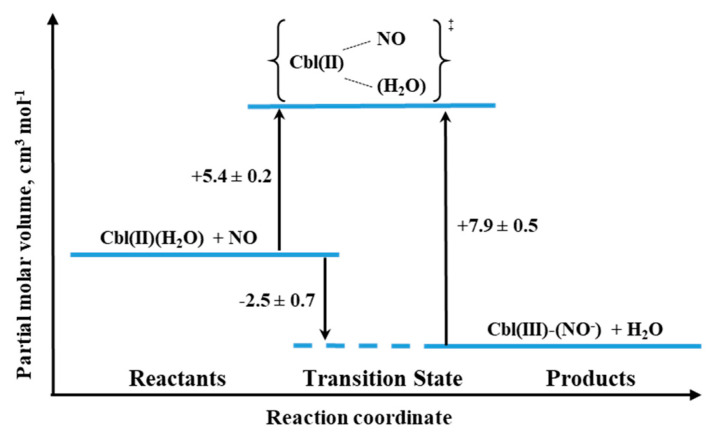
The volume profile for the reaction of Cbl(II) with NO in aqueous solution at pH = 7.4.

Based on NMR studies, the actual form of the final product was defined as Cbl(III)(NO^−^), in agreement with the products of reductive nitrosylation of Cbl(H_2_O) and Co(III) porphyrins. The activation parameters were determined for both the formation and decay of the nitrosyl complex by using laser flash photolysis and stopped-flow techniques, respectively. The effect of pressure was studied for both reactions. The small positive value of ΔV^‡^_on_ for Cbl(III)(NO^−^) formation (+5.4 cm^3^/mol at pH = 7.4) suggested implementation of the dissociative interchange mechanism, which was possible due to the appearance of a water-bound intermediate. A similar type of mechanism is being realized in the reverse reaction forced by the use of NO-trapping technique, as indicated by the value of ΔV^‡^_off_ = +7.9 cm^3^/mol. The volume profile for the reaction 11 is shown in Figure 20.

One of the biologically relevant pathways for the interaction of Cbl(H_2_O) with NO involves reactions with endogenous S-nitrosothiols (RSNO). Their binding to the metal center, regardless of whether realized through a sulfur or nitrogen atom, usually leads to homolytic cleavage of the S-NO bond, which may affect nitric oxide homeostasis. This prompted an in-depth study on the mechanisms of these interactions, considering various potential reaction sequences leading to the formation and further fate of Cbl(RSNO). Application of high-pressure techniques significantly contributed to the elucidation of the mechanism of the reaction of glutathionylcobalamin (CblSR) with NO. The negative value of ΔV^‡^ (−9.6 cm^3^/mol) indicates an associative reaction pathway, leading to the formation of S-nitrosoglutathionylcobalamin. In the following rapid step, it decomposes to GSNO and the reduced cobalt complex [61]:(12)
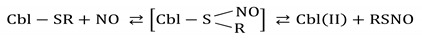


A similar mechanistic scenario has been observed for nitrosylation of thiolato ligand in the model Fe(III)–thiolate porphyrin complex. In this case, high-pressure kinetic studies also revealed negative ΔV^‡^_on_ values viz. −16.3 cm^3^/mol pointing towards a common associatively activated reaction pathway [32].

In recent years, another Reactive Nitrogen and Oxygen Species (RNOS), namely HNO, has been receiving increasing attention, due to the finding of its unique—and, in some cases, even complementary to NO—function in biological systems [62]. In order to thoroughly explore the mechanism of the reaction of HNO with Cbl(H_2_O), kinetic studies were performed over a wide pH range using Piloty’s acid (PA) as the nitroxyl donor [63]. Significant differences regarding, among others, the rate-determining step of Cbl(III)(NO^−^) formation were found depending on the PA concentration. The results of the kinetic studies revealed the pK_a_ of nitroxyl to be 11.4. Following the pH scale, particularly interesting observations were made in the neutral range, where the reaction product is formed remarkably fast despite the high stability of PA. This unusual behavior was explained on the basis of the formation of the Cbl(III)(PA) intermediate, which decays to nitrosylcobalamin upon deprotonation and S–N bond breaking. High-pressure techniques have been employed to confirm this mechanism. The near-zero ΔV^‡^ value (+1.4 cm^3^/mol at pH = 6.1) indicates that there are no significant changes in the partial molar volumes during the conversion step of Cbl(III)(PA) to the final product.

## 6. Conclusions

High-pressure techniques for kinetic and mechanistic studies represent a unique and irreplaceable tool in the elucidation of reaction mechanisms in solution. The information provided by the activation volume makes it possible to unambiguously determine the nature of the underlying reaction mechanism, which gives an advantage over the activation entropy obtained by analyzing the effect of temperature. Despite the technical complexity, due in part to the relatively slow establishment of equilibrium in solution after a change in applied hydrostatic pressure, it is possible to follow the course of reactions occurring over a wide range of timescales by combining the high-pressure technique with standard, stopped-flow, and relaxation (laser flash photolysis and pulse radiolysis) techniques. This is particularly useful for studying fast inorganic reactions, which, in solution, are often applicable to biological systems.

In this review, we have attempted to demonstrate the extent to which high-pressure studies have influenced the understanding of the mechanisms of NO interactions with biologically active metal centers. The redox character of these reactions, due to both the radical nature of NO and the availability of various oxidation states of metals (iron and cobalt), makes the results of pressure studies unique in providing more parameters of the complex formed than just the composition of the coordination sphere. Systematic studies extending from simple iron complexes, through Fe(II) and Fe(III) porphyrins, to protein complexes of heme (myoglobin and cytochrome P450), have demonstrated different abilities of the oxidized and reduced metal species to bind NO. Of similar importance was the need to prove the influence of ligands—in particular, the axial ligand on porphyrin complexes—in these reactions. The mechanistic information obtained from the analyses of volume profiles, and especially the position of the transition state on the volume profile, reveals new mechanistic information that was not available before. The possibility to visualize the chemical process in terms of partial molar volume changes is rather unique and opens up further theoretical work based on such volume changes. Characterization of the transition state by changing the partial molar volumes contributed to the demonstration of a wide variety of spin states, bond lengths, and electron density distributions. High-pressure studies focused on cobalt complexes—although few in number—have provided important arguments in distinguishing the biological role of Co and Fe. Complete characterizations of the intermediates in the nitrosylation reactions of both Co(II) and Co(III) porphyrins and reduced vitamin B12 are among the most important results of these studies.

We are therefore fully convinced that this currently somewhat marginalized field of chemistry possessing such a powerful research tool still has much to offer.

## Figures and Tables

**Table 1:** Rate constants and activation volumes for the reactions of selected iron porphyrins and heme proteins with NO.

	*k*_on_ (M^−1^ s^−1^)	ΔV^‡^_on_ (cm^3^/mol)	*k*_off_ (s^−1^)	ΔV^‡^_off_ (cm^3^/mol)	Reference
Fe^III^(TPPS^4−^)(H_2_O)_2_	4.5 × 10^5 (at 25 °C)^	+9 ± 1	5 × 10^2 (at 25 °C)^	+18 ± 2	[26]
Fe^III^(TMPS^4−^)(H_2_O)_2_	2.8 × 10^6 (at 25 °C)^	+13 ± 1	9 × 10^2 (at 25 °C)^	+17 ± 3	[26]
Fe^III^(TMPS^4−^)(OH)	7.4 × 10^3 (at 10 °C)^	−16.2 ± 0.4	1.5 ^(at 10 °C)^	+7.4 ± 1.0	[27]
Fe^III^(P^8−^)(H_2_O)_2_	8.2 × 10^5 (at 25 °C)^	+6.1 ± 0.1	2 × 10^2 (at 25 °C)^	+16.8 ± 0.4	[28]
Fe^III^(P^8−^)(OH)	5.1 × 10^4 (at 25 °C)^	−6.1 ± 0.2	11 ^(at 25 °C)^	+17 ± 3	[28]
Fe^III^(P^8+^)(H_2_O)_2_	1.5 × 10^4 (at 25 °C)^	+1.5 ± 0.3	26 ^(at 25 °C)^	+9.3 ± 0.5	[29]
Fe^III^(P^8+^)(H_2_O)(OH)	1.6 × 10^3 (at 25 °C)^	−13.8 ± 0.4	6.2 ^(at 25 °C)^	+2.6 ± 0.2	[29]
Fe^II^(TPPS^4−^)(H_2_O)_2_	1.5 × 10^9 (at 25 °C)^	+5 ± 1	-	-	[26]
Fe^II^(TMPS^4−^)(H_2_O)_2_	1.0 × 10^9 (at 25 °C)^	+2 ± 1	-	-	[26]
metMb	4.8 × 10^4 (at 25 °C)^	+21 ± 1	29 ^(at 25 °C)^	+16 ± 1	[30]
P450_cam_—resting state	3.2 × 10^5 (at 25 °C)^	+28 ± 2	0.35 ^(at 25 °C)^	+31 ± 1	[31]
P450_cam_—camphor	3.2 × 10^6 (at 25 °C)^	−7.3 ± 0.2	1.93 ^(at 25 °C)^	+24 ± 1	[31]
SR complex in methanol	2.7 × 10^6 (at 25 °C)^	+6 ± 1	1.8 ^(at 25 °C)^	-	[32]
R-SO_3_^−^ complex in methanol	6 × 10^4 (at 25 °C)^	−21 ± 4	2.2 × 10^3 (at 25 °C)^	+7 ± 3	[33]
R-SO_3_^−^ complex in toluene	1.8 × 10^6 (at 25 °C)^	−25 ± 4	1.2 × 10^4 (at 25 °C)^	+7 ± 3	[33]

**Table 2:** Summary of activation-volume data for the reversible coordination of NO by a series of Fe^II^(L) complexes, where L represents a series of polyaminocarboxylate complexes.

Complex	ΔV^‡^_on_ (cm^3^/mol)	ΔV^‡^_off_ (cm^3^/mol)	Reference
[Fe^II^(H_2_O)_6_]^2+^	+6.1 ± 0.4	+1.3 ± 0.2	[44]
[Fe^II^(edta)(H_2_O)]^2+^	+4.1 ± 0.2	+7.6 ± 0.6	[37]
[Fe^II^(hedtra)(H_2_O)]^2+^	+2.8 ± 0.1	+4.4 ± 0.8	[37]
[Fe^II^(nta)(H_2_O)_2_]^2+^	−1.5 ± 0.1	−3.5 ± 0.7	[37]
[Fe^II^(mida)(H_2_O)_3_]^2+^	+7.6 ± 0.4	+6.8 ± 0.4	[37]
[Fe^II^(mida)_2_(H_2_O)]^2+^	+8.1 ± 0.2	+5.1 ± 0.5	[37]
[Fe^II^(CN)_5_(H_2_O)]^3−^	+17.4 ± 0.3	+7.1 ± 0.2	[39]
